# Examining the vulnerability of adult neuron subtypes to tau-mediated toxicity in *Drosophila*

**DOI:** 10.1038/s41398-025-03342-2

**Published:** 2025-04-05

**Authors:** Lovesha Sivanantharajah, Amrit Mudher, David Shepherd

**Affiliations:** 1 School of Environmental and Natural Sciences, Bangor University, Bangor, Gwynedd UK; 2 Faculty of Natural and Environmental Sciences, University of Southampton, Southampton, UK

**Keywords:** Diseases, Neuroscience

## Abstract

Selective vulnerability of nerve cells is a feature of neurodegenerative disease. To date, animal models have been limited to examining pathogenic protein expression in broad or heterogeneous neuronal populations. Consequently, noted pathological hallmarks represent an average of disease phenotypes over multiple neuron types, rather than exact measures of individual responses. Here we targeted gene expression to small, precisely defined and homogenous neuronal populations in the *Drosophila melanogaster* central nervous system (CNS), allowing dissection of selective vulnerability of single types of neurons with *single-neuron resolution*. Using cellular degeneration as a readout for vulnerability, we found while all neurons were affected by tau some neuron types were more affected (vulnerable) than others (resilient). The tau-mediated pathogenic effects fell on a spectrum, demonstrating that neurons in the fly CNS are *differentially vulnerable* to tau pathology. Mechanistically, total tau levels did not correlate with vulnerability; rather, the best correlatives of degeneration were significant age-dependent increases in phospho-tau levels in the same neuron type, and tau mislocalisation into dendrites. Lastly, we found that tau phosphorylation in vulnerable neuron types correlated with downstream vesicular and mitochondrial trafficking defects. However, all vulnerable neuron types did not show the same pattern, suggesting multiple paths to degeneration. Beyond highlighting the heterogeneity of neuronal responses to tau in determining vulnerability, this work provides a new, high-resolution, tractable model for studying the age-dependent effects of tau, or any pathogenic protein, on postmitotic neurons with sub-cellular resolution.

## Introduction

Neurodegenerative disorders share two characteristics: accumulation of pathological proteins and progressive selective deterioration of specific neuronal subtypes. Tauopathies, like Alzheimer’s Disease (AD), are marked by neuronal aggregation of hyper-phosphorylated tau, a microtubule associated protein (MAPT) required for microtubule stability [1–3]. Typically, progressive deterioration of neurons in AD begins in brain regions involved in memory before progressing to connected cortical areas [4, 5]. This is interesting because tau expression is ubiquitous across the brain. That tau does not become pathogenic in all neurons, strongly indicates that different cell types are *selectively* at risk for developing tau pathology. Thus, understanding why all neurons are not equally susceptible to pathogenic tau is essential for identifying mechanisms underpinning disease pathobiology, and developing new treatment strategies.

Selective neuronal vulnerability to disease is complex, resulting from many intrinsic and extrinsic factors [6]. The ability to collectively study multiple deterministic factors pre-symptomatically has posed a real problem for dissecting selective vulnerability; however, animal models of disease like *Drosophila* have proven invaluable for recapitulating human disease symptoms in a low cost and genetically tractable system [7]. Fly tauopathy models demonstrate that selective vulnerability is multi-faceted, involving differential distribution of tau-modifying kinases and phosphatases across different neuronal classes [8], and the importance of primary neurotransmitter systems [9]. However, many previous studies analysed tau expression in heterogenous classes of neurons, where pathogenic phenotypes represent an average of disease phenotypes over several cell types, and do not suppress tau expression during development rendering it difficult to separate developmental tau phenotypes from those acquired over lifespan [7].

Addressing these issues, we used a collection of *Drosophila* lines using the Split-GAL4 intersectional strategy to target gene expression to small, precisely defined classes of neurons in the *Drosophila* ventral nervous system (VNS) [10, 11]. These neuronal populations represent single-neuron types, part of developmental lineages produced by single neuronal precursor cells, and represent functional modules within the central nervous system (CNS) serving specific behavioural functions and sharing common attributes of morphology, transcriptional profiles and neurotransmitter type [12–14]. This refined toolset offers unprecedented resolution to study tauopathy. Temporally, driver lines restrict transgene expression to postmitotic adult neurons. Spatially, tau expression is narrowly restricted to single-neuron types. Further, the small number of labelled neurons (4–20 cells) allows us to resolve subcellular structures and compartments. Lastly, tau expression within specific neurons, in an otherwise normal nervous system, ensures we are analysing primary pathological events only.

Using a subset of available genetic lines, we undertook an unbiased screen of the effects of the highly-phosphorylated human tau isoform, hTau^0N3R^, to identify neuron types demonstrating vulnerability or resilience to tau as defined by unambiguous criteria: the degree and extent of cellular degeneration. Our model recapitulated previously reported typical age-dependent tau phenotypes, including loss of synaptic terminals, tau mislocalisation into dendrites, and beading and blebbing of neuronal processes containing tau accumulates [15, 16]. We found tau expression in different neuron types produces a spectrum of pathological effects, demonstrating that fly VNS neurons are *differentially vulnerable* to tau pathology. Examination of tau-dependent pathogenic mechanisms (e.g., localisation and phosphorylation) and mechanisms downstream of tau (e.g., vesicular and mitochondrial trafficking) highlight the heterogeneity of neuron type specific responses to tau when examined at single-cell resolution.

## Methods

### Fly genetics and maintenance

SplitGAL4 driver lines (Fig. S1; Table S1) drove expression of the highly-phosphorylated and toxic human tau isoform, 0N3R (hTau^0N3R^), in postmitotic neurons in the adult *Drosophila melanogaster* VNS (Janelia FlyLight Project, [11]). Eight neuron types were selected from a larger screen of 24 for having clear, tau-mediated phenotypes without problematic GAL4 driver expression issues (e.g., no larval brain expression (Fig. S2), all drivers on in pupal brains at stage P8 (yellow-eyed, [17]) of metamorphosis (Fig. S3), and all drivers showing continued expression past 3wks post-eclosion). Crosses were maintained at 18 °C and adults moved to 25 °C post-eclosion for aging. Flies expressing LacZ β-Galactosidase (LacZ, Exelixis, Inc.) were used as controls for protein overexpression. Neuron morphology was revealed by co-expression of membrane-bound GFP (UAS-mCD8GFP). For mechanistic analysis of tau pathology, hTau^0N3R^ or LacZ were co-expressed with the UAS-reporter lines, mitoGFP, for labelling mitochondria [18], and nSyb-GFP, for labelling synaptic vesicles transported along axons to synaptic terminals [19].

### Tissue preparation and immunohistochemistry

Adult female VNSs were dissected at 0 (0wks) and 21 days (3wks) post-eclosion. Dissections, tissue preparation and immunostaining were performed as previously detailed [20]. Primary antibodies used were: (1:1000) chicken anti‐GFP (Invitrogen-Molecular Probes, Cat. no. A10262), (1:15,000) rabbit anti‐hTau (Agilent- DAKO, Cat. no. A002401-2), (1:125) mouse anti-LacZ (J1E7, DSHB); (1:1000) mouse anti-phospho-Tau (Ser202, Thr205) antibody (AT8, Fisher, Cat. no. 10599853). Relevant secondary antibodies were used at (1:500). Images were acquired with a Zeiss LSM710 confocal microscope. For mechanistic analyses, identical image acquisition parameters were used.

### Blind scoring of phenotypic severity

Neuronal morphology was scored blind to treatment (e.g., with or without hTau^0N3R^ and timepoint, *n* = 3–10 per group) [15]. Neurons were scored from 0 (no affect) to 3 (highly affected) using the presence or absence of previously described tau-mediated phenotypes (Criteria in Table S2). Average Severity Score was calculated for each group.

### Data and statistical analyses

Images were contrast‐enhanced as necessary and analysed in ImageJ (http://rsbweb.nih.gov/ij/). Fluorescence intensity (i.e., levels) is displayed as Corrected total cell fluorescence (CTCF), where CTCF = Integrated Density - (Area of selected cell x Mean fluorescence of background readings) [21, 22]. Mitochondrial number and volume were analysed using Imaris v10.0.1 (Oxford Instruments). Statistical analyses were performed with GraphPad Prism v9.1.0 (GraphPad Software, Inc., San Diego, CA). Statistical tests were selected based on data meeting requirements for homoscedasticity and normality for use of parametric tests; where requirements were not met, non-parametric tests were used. See Table S3 for details of statistical tests used and results of all comparisons. Samples were not analysed in a randomized manner. No statistical methods were used to determine sample size. No animals were excluded from analysis. No blinding was used in our assays except in blind scoring of phenotypic severity across neuron types.

## Results

### Neuronal morphology reveals single-neuron types are differentially vulnerable to tau pathology

**Fig. 1:**
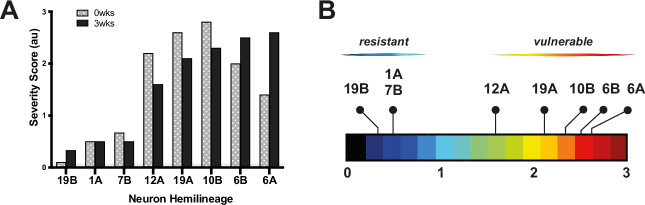
Neuron types in the *Drosophila* ventral nerve cord are differentially vulnerable to tau-mediated toxicity. **A** Graph of morphological severity scores from blind scoring of images at 0- and 3-wks post eclosion. hTau^0N3R^ was expressed in different neuron types and blind scoring of cellular morphology was used to determine neuron vulnerability to hTau^0N3R^-mediated phenotypes. **B** Neuronal vulnerability to tau-mediated toxicity is a spectrum, with some neuron types being relatively more affected (vulnerable types) than others (resilient types). The most resilient neuron type was 19Bβ, whereas 6A was the type most vulnerable to tau-mediated toxicity.

To study the impact of tau expression on neuronal morphology, the 0N3R isoform of human tau (hTau^0N3R^) was co-expressed with membrane bound GFP (mCD8-GFP). The particular hTau^0N3R^-expressing genetic line used here was chosen as our lab have extensively studied this isoform and shown it to be constitutively highly phosphorylated in vivo and found that it induces visible degenerative phenotypes [23–25]. Since each neuron type has stereotypical morphology, anatomical changes (shape, size, membrane blebbing, dendritic and synaptic morphology/loss) can be easily detected. Detailed anatomical descriptions for neuron subtypes examined here are published [20] (Table S1). Our model recapitulated typical age-dependent tau phenotypes including loss of synaptic terminals, mislocalisation of tau into dendrites, and beading and blebbing of neuronal processes containing tau accumulates [15, 16]. Blind Scoring (Fig. 1A) of morphology found that whilst tau expression impacted all neurons, the severity of effects represents a spectrum of pathology with some more affected than others (Fig. 1B). Despite differences across neuron types, responses of the same neuron type were consistent across replicates, exhibiting comparable defects over the same timeframe. Generally, tau expressing neurons fell into two broad categories: (i) *resilient* neurons exhibiting only minor defects at 0wks without significant progressive deterioration by 3wks (neurons types: 1A, 7Bα, 19Bβ), and (ii) *vulnerable* neurons exhibiting early onset and significant defects (neuron types: 10Bβ, 12A, 19Aα, 6A and B). Vulnerable neurons demonstrated either an age-dependent worsening of tau-mediated phenotypes over time (6A, B) or an early severe phenotype at 0wks that plateaued by 3wks (10Bβ, 12A, 19Aα).

#### Most vulnerable type, 6A

6A neurons are GABAergic components of the circuits regulating sensory feedback during flight [12, 14]. Our driver labels 6A only in the prothoracic neuromere (t1). In t1, 6A neurons project into dorsal neuropil forming extensive, primarily dendritic arborisations before sending a primary axonal projection into t2 to synapse onto downstream targets (Table S1). Here, 6A neurons were the most severely affected with the highest average degenerative score at 3wks.

**Fig. 2:**
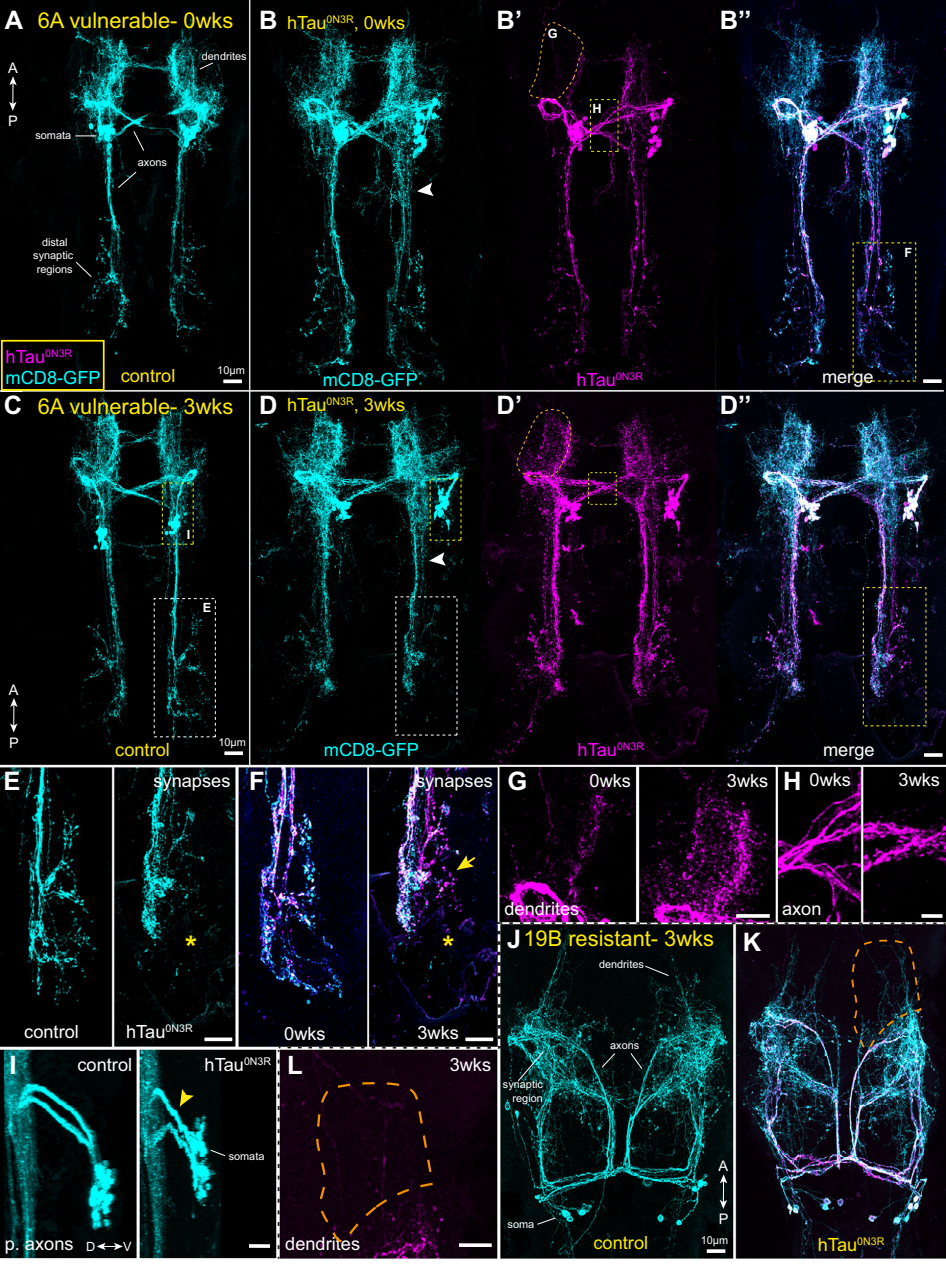
hTau^0N3R^ expression in the vulnerable 6A neurons causes degeneration in 3wk old adult flies, but not in the most resilient 19Bβ neurons. **A** Control 6A neurons at 0wks appear normal. **B** hTau^0N3R^-expressing 6A neurons at 0wks show some moderate signs of degeneration, including defasciculation of axonal processes (arrowhead). **C** Control 6A neurons at 3wks show some signs of age-dependent degeneration like axonal defasciculation but are otherwise normal. D. hTau^0N3R-^expresing 6A neurons at 3wks however show obvious neuronal degeneration. These neurons exhibit many of the classic features of tau toxicity: degeneration of defasciculated axons (**D**, arrowhead), axon thinning and fragmented synaptic terminals **E**, extracellular tau where cellular membranes have degenerated at 3wks (**F**, arrow), tau mislocalisation into dendrites over time **G**, and tau accumulation and change in appearance from continuous to beaded/fragmented over time **H**, shortening of and swellings in proximal axonal compartment (**I**, yellow arrowhead), Asterisks (*) highlight loss of terminal synaptic regions at 3wks. By contrast, age matched resilient 19Bβ neurons **J,** **K** show little evidence of these features. Neuron morphology between control **J** and hTau^0N3R^ expressing **K** neurons are not dissimilar. hTau^0N3R^ is confined to large axonal compartments and there is little to no tau mislocalisation into dendritic compartments **L**.

At 0wks, control neurons (Fig. 2A) appear normal and hTau^0N3R^-expressing neurons (Fig. 2B) show some axon defasciculation (Fig. 2B, arrowhead) and other moderate early signs of degeneration, including membrane swellings in axons, dendrites and synaptic boutons. At 3wks, control 6A neurons (Fig. 2C) generally appeared normal with only mild age-related phenotypes including some fragmentation in dendrites and axons and some axon defasciculation. In contrast, hTau^0N3R^-expressing 6A neurons (Fig. 2D) showed severe morphological degeneration including fragmentation of defasciculated axons (Fig. 2D, arrowhead), axon thinning and fragmented synaptic boutons with loss of distal synaptic regions (Fig. 2E, asterisk), and shortening and swelling of proximal axonal compartments (Fig. 2I, yellow arrowhead).

#### Most resilient type, 19Bβ

19Bβ neurons are cholinergic premotor flight interneurons [12, 14]. Our driver labels only 19Bβ neurons in the mesothoracic neuromere (t2). In t2, 19Bβ neurons enter the neuropil dorsally at the posterior margin of the neuromere and project medially crossing the midline at the posterior margin of the t2 neuropil (Table S1). With the exception of a small dendritic region, the synaptic region of this neuron type is axo-dendritic, meaning a mix of inputs and outputs. Here, 19Bβ neurons were least affected with the lowest average degenerative severity scores at both 0- and 3wks. At 0wks, hTau^0N3R^-expressing neurons looked normal, resembling controls, with little sign of any of the morphological changes described for vulnerable neurons. At 3wks, all control and tau-expressing neurons continued to look normal (Fig. 2J, K), with the exception of single outliers in both categories: one control neuron showed possible signs of axonal degeneration and one tau-expressing neuron showed some abnormal tau accumulation in fine processes. This data shows that the resilient neurons are resistant to developing pathology following expression of human tau, even if the tau has been expressed for several weeks.

### Tau-dependent mechanisms for neuronal vulnerability

#### Appearance and cellular localization of tau

Generally, hTau^0N3R^ accumulated over time (0- vs. 3wks) in all neuron types examined and its distribution throughout the neuron changed with time with tau moving from axons into more distal and finer neuronal processes. However, two common features were associated with vulnerability: (i) greater accumulation of tau within fine processes and mislocalisation into dendrites over time and (ii) a change in the appearance over time of hTau^0N3R^ from smooth and continuous to a fragmented form that is usually indicative of abnormal tau accumulation [15]. The fragmented appearance of tau alone was not correlated with degeneration if tau was confined to the main processes. However, the appearance of tau in more finer processes, often in aged neurons, was correlated with greater degeneration throughout the entire neuron in regions where tau is more fragmented.

In the most vulnerable 6A neurons at 0wks (Fig. 2B’) there were low levels of hTau^0N3R^ tau in dendrites (Fig. 2F), and some beaded tau in long axons although most tau is smooth and continuous (Fig. 2H). At 3wks, hTau^0N3R^ in 6A neurons (Fig. 2D’) had mislocalised into dendrites and the appearance of tau in this compartment is small and fragmented (Fig. 2F). In the proximal axonal compartment, tau has changed over time from smooth and continuous to fragmented in appearance (Fig. 2H). In addition to visible loss of the most distal pre-synaptic regions (Fig. 2F, asterisk), this region also contains “extracellular” tau which are neuronal structures that are hTau positive but mCD8GFP negative (Fig. 2F, arrow) and thus resemble ghost tangles [26].

In the least affected type, 19Bβ, tau appears as small, beaded aggregates at 0wks confined to larger axon segments and rarely observed in finer processes. At 3wks, tau aggregates remained mainly in larger axon segments, with little mislocalisation of tau into finer dendritic processes (Fig. 2L). Although tau is present in this neuron type and is beaded in some places, it is largely smooth in appearance and does not take on a beaded fragmented appearance. These neurons remain morphologically normal, looking like controls even at 3wk (Fig. 2K).

In other resilient types, a similar pattern of tau distribution is observed. In 1A neurons small accumulates of tau are restricted to the main axons with little in dendrites and finer processes at both 0 and 3wks. A correlation between the presence of fragmented tau and morphological degeneration is seen in 7Bα neurons classified as “resilient” but showing some insightful intermediary phenotypes. At 0wks, tau in 7Bα is fragmented but is confined to the main processes with neuron membranes showing some signs of fragmentation in finer processes. At 3wks, there is a variable phenotype where in neurons with generally normal morphology, tau is smooth and continuous in long axons with some fragmentation in finer processes (Figure S5). When tau is fragmented throughout both long axons and finer processes there are early signs of degeneration. In such 7Bα neurons, localised degeneration is seen and correlated with the colocalization of beaded/fragmented tau.

#### The severity of morphological phenotypes does not correlate with total hTau levels

**Fig. 3:**
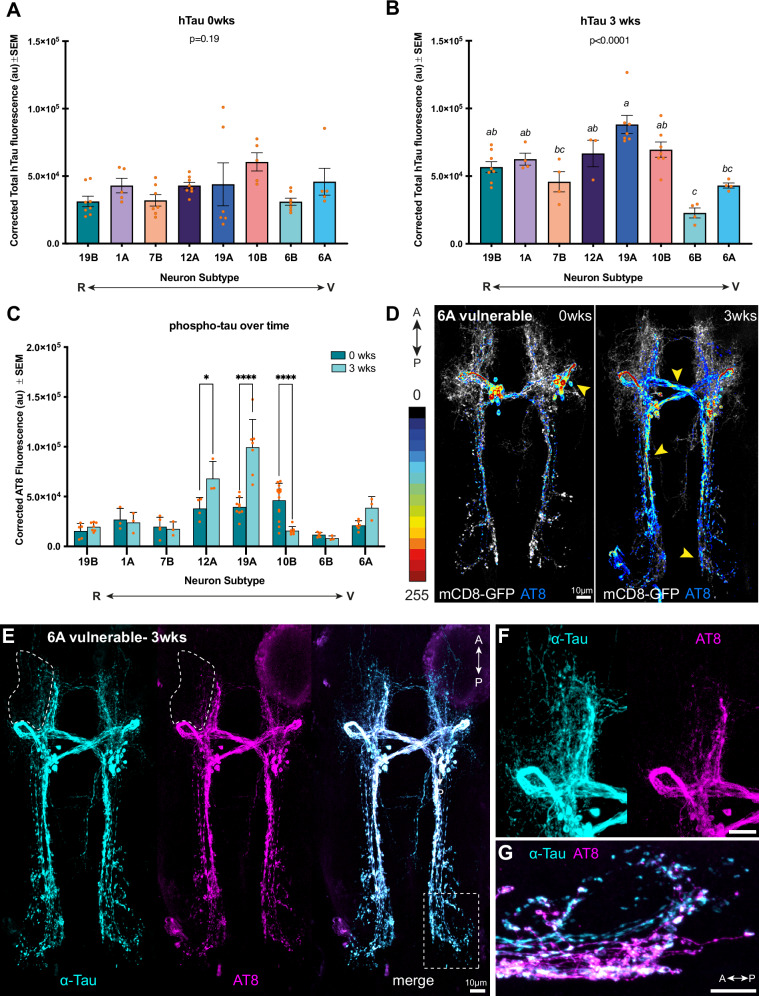
Tau dependent mechanisms for vulnerability. An increase in phosphorylated tau (p-tau) levels, but not total tau, over time correlates with neuronal vulnerability. **A** Levels of total tau between all neuron subtypes does not significantly differ at 0wks (*P* = 0.19)(*n* = 5–8 per group). **B** At three weeks there is a significant difference in tau levels between neuron subtypes (*P* < 0.0001)(*n* = 3–8 per group); however, there is no clear correlation between phenotypic severity and tau levels across resilient or vulnerable neuron types. Data with the same letters are not significantly different (*P* > 0.05). **C** There are significant differences in p-tau (AT8) levels at 3wks compared to 0wks for some vulnerable neuron subtypes, but not resilient types. The vulnerable neurons, 12 and 19A, show a significant increase in p-tau levels over time; however, 10B shows a significant decrease and 6B no change. Whilst 6A neurons show a trend towards increase of p-tau levels over time, this is not significant post correction for multiple comparisons (*P* = 0.38)(*n* = 3–14 per group). Asterisks denote significance as follows: *P < 0.05; ****P < 0.0001. **D** Maximum intensity projections (MIPs) of p-tau expression in vulnerable 6A neurons at 3wks are shown as heatmaps where pixel intensity is correlated with a colour scale. Over time, p-tau expression can visually be seen to accumulate and spread to more distal neuronal compartments in the vulnerable neuron type 6A. Arrowheads indicate hotspots of p-tau accumulation. Where p-tau is acutely localised to soma and proximal axons at 0wks, there are many more p-tau hotspots in distal axons and presynaptic areas at 3wks. **E** Phospho-tau labels a subset of total tau (DAKO). Regions within perforated lines highlight differences in total tau and p-tau localisation in finer processes, which have been magnified in **F,** **G**. **F** Higher magnification images of the dendrites showing that hTau^0N3R^ has spread into the dendrites at 3wks; however, there is little p-tau spread (AT8). **G** Higher resolution image of 6A distal axon terminals and pre-synaptic regions show p-tau has not penetrated the most distal cellular regions.

To determine if morphological severity across neuron types was the result of varying hTau^0N3R^ expression levels, we quantified hTau^0N3R^ levels at 0- and 3wks in all eight neuron types. At 0wks all neuron types had comparable levels of hTau^0N3R^ expression [F(7,13.61) = 1.704; *P* = 0.19] (Fig. 3A). At 3wks there were significant differences in total hTau^0N3R^ levels between neuron types [F (7,33) = 11.37; *P* < 0.0001]; however, post-hoc Tukey analysis found no clear relationship between phenotypic severity and total tau levels (Fig. 3B). To further investigate the relationship between total tau levels and degeneration, degenerative severity scores for resilient and vulnerable neurons at 3wks was compared with total hTau^0N3R^ levels at 3wks. This also showed no correlation (r = −0.10, *P* = 0.80) suggesting degenerative severity is not caused by hTau^0N3R^ levels (Fig. S4). This is evident when comparing tau levels across resilient and vulnerable neuron types; for example, hTau^0N3R^ levels are significantly greater in resilient 19Bβ neurons than in vulnerable 6B neurons (Fig. 3B).

#### Vulnerable neurons generally show significant age-dependent increase in hTau phosphorylation

With total tau levels not appearing to be the cause of vulnerability, we next explored the role of tau phosphorylation in differential vulnerability as several studies, including those from our lab, have previously implicated tau phosphorylation in tau toxicity [23, 27]. We examined tau phosphorylation at the AT8 epitope site (pS202/pT205) in hTau^0N3R^, a site associated with AD pathology [28–30]. We examined phospho-tau levels at AT8 sites (p-tau) across our eight neuron times at two timepoints (0 and 3-wks post-eclosion). A two-way ANOVA was used to analyse the effects of neuron-type and time on p-tau levels (i.e., corrected AT8 fluorescence), and found a significant interaction between neuron-type and time (F (7, 71) = 16.36, *P* < 0.0001). To examine the specific relationships within this interaction more closely, Simple Main Effects Analysis was used and found that neuron-type has a statistically significant effect on p-tau levels (*P* < 0.0001), and that time has a significant effect on p-tau levels (*P* = 0.0042). To better understand which of these factors could best explain our noted neuronal vulnerability, further analysis of this complex data was carried out. Whilst different neuron types at a single timepoint show many significant differences in their p-tau levels (Fig. S6A, B), p-tau levels do not correlate with time-matched morphological severity scores at either 0wks (r = 0.69, *P* = 0.06) (Fig. S6C) or 3wks (r = 0.23, *P* = 0.58) (Fig. S6D). Thus, neuron type at a particular time point cannot provide an explanation for the observed vulnerability. However, post-hoc analysis of p-tau levels for the same neuron type across time (0wks vs. 3 wks), found that resilient types (1A, 19Bβ or 7Bα) do not show significant changes over time, but some vulnerable neurons do (Fig. 3C). 19 and 12A neurons show significant increases and 10B a significant decrease. 6A neurons show an increase in p-tau levels that is not significant post correction for multiple comparisons. Thus, the change in p-tau levels over time for the same neuron type provides some explanation for the observed vulnerability.

A visual examination of p-tau accumulation and localisation in the vulnerable 6A neurons, shows how p-tau accumulates first in cell bodies and proximal axons at 0wks before accumulating and localising to more distal regions with time (Fig. 3D). However, p-tau labelled only a subset of total hTau immunoreactivity, being notably absent from finer neuronal processes of dendritic compartments and the most distal synaptic regions (Fig. 3E–G). In the most vulnerable 6A neurons, whilst there is a significant amounts of total tau at 3wks in the dendritic compartment, very little of it is p-tau positive (Fig. 3F). In the pre-synaptic regions of this neuron, p-tau is present but unlike total-tau is not present in the finer, most-distal processes (Fig. 3G).

This data collectively implies that it is not the amount of tau within a neuron but its accumulation of p-tau with time that plays a role in determining its vulnerability to degeneration.

### Mechanisms downstream of tau contributing to vulnerability

#### Vesicular trafficking

**Fig. 4:**
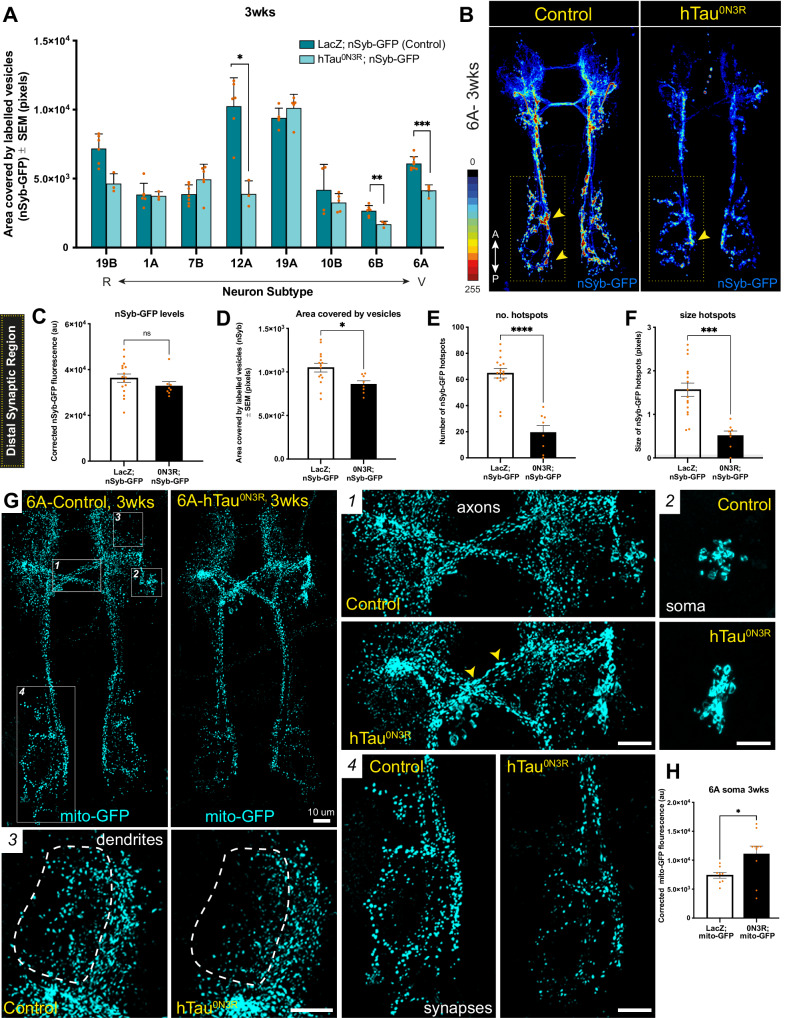
Neurons that show vesicular trafficking defects are vulnerable types. **A** The vulnerable neurons 12A, 6B and 6A all show significantly decreased area covered by GFP labelled synaptic vesicles (nSyb-GFP) in tau expressing neurons compared to control at 3wks. This suggests the presence of trafficking defects in these neuron types when hTau^0N3R^ is present. (*n* = 3–8 per group) **B** Maximum intensity projections (MIPs) of nSyb-GFP expression in vulnerable 6A neurons at 3wks are shown as heatmaps of pixel intensity correlated with colour scale for control (LacZ-expressing) and hTau^0N3R^-expressing neurons. Heatmaps of nSyb-GFP expression in 6A neurons reveal that whilst there are evenly distributed hotspots of nSyb-GFP accumulation (red) in control neurons, these are lacking in hTau^0N3R^-expressing neurons. Yellow boxes highlight the distal pre-synaptic regions which most clearly show differences in nSybGFP localisaion, and arrowheads point to hot spots. **C**–**F** Closer examination of the most distal pre-synaptic regions of 6A neurons (regions enclosed by yellow boxes in B) found that whilst nSyb-GFP levels do not change between control and hTau^0N3R^ expressing neurons (**C**), there is a significant decrease in area covered by nSyb-GFP when tau is overexpressed (**D**)(*n* = 8–16 per group). More specifically this can be explained by significant reductions in the number (**E**) and size (**F**) of nSyb-GFP hotspots in the distal pre-synaptic regions of hTau^0N3R^-expressing neuron (*n* = 8–16 per group). **G** Mitochondrial trafficking at 3wks in control and hTau^0N3R^-expressing vulnerable 6A neurons was visualised using mito-GFP to label mitochondria. Enlarged regions of control and tau-expressing neurons are shown for axons (1), soma (2), dendrites (3) and synapses (4) of 6A neurons at 3wks. Where control neurons show similarly shaped mitochondria that are evenly distributed, hTau^0N3R^-expressing neurons show evidence of trafficking defects. Arrowheads highlight irregularly shaped mitochondria and clumping of mitochondria in axonal regions (2). There are fewer mitochondria in the dendrites (3) and distal synaptic regions (4) of tau-expressing neurons and mitochondria accumulate in the soma (2). **H** There is a significant increase in the amount of mitoGFP that accumulates in the soma of hTau^0N3R^ expression 6A neurons compared to controls at 3wks, which is consistent with mitochondrial trafficking defects (*n* = 8–10 per group). Statistical difference is noted with asterisks as follows: ns, not significant; *P < 0.05; **P < 0.01; ***P < 0.001.

Accumulation of synaptic vesicles along axons is a hallmark of defective axon transport [23]. Therefore, the effects of hTau^0N3R^ on vesicular trafficking were studied by examining area covered by GFP-labelled synaptic vesicles (nSyb-GFP) across individual neuron-types to determine if this area changed when compared to control neurons expressing the innocuous protein, LacZ (control). Given that neuron size can vary, only measures of area (control versus hTau^0N3R^) for a neuron-type were compared using unpaired t-tests. Comparisons for all eight neurons at 3wks are plotted on the same graph with neuron type arranged from most resilient to most vulnerable on the x-axis to help visualise any relationship with vulnerability (Fig. 4A). At 3wks, resilient neuron types (19Bβ, 1A, 7Bα) showed no significant differences in area covered by GFP-labelled vesicles between neurons expressing LacZ or hTau^0N3R^, after correcting for multiple comparisons (Fig. 4A). In vulnerable neurons, 12A (t_(7)_ = 4.9; *P* = 0.002), 6B (t_(7)_ = 4.1; *P* = 0.005) and 6A (t_(10)_ = 6.8; *P* < 0.0001) showed significant decreases in area covered by labelled vesicles when hTau^0N3R^ was present. 10Bβ and 19Aα neurons showed no difference.

Closer examination of vesicular trafficking in vulnerable 6A neurons at 3wks found that while total levels of GFP-labelled vesicle across the entire neuron were not different between control and hTau^0N3R^-expressing neurons (t_(10)_ = 1.8; *P* = 0.11)(Fig. S7C), heatmaps of maximum intensity projections (MIP) of GFP-labelled vesicle expression reveal evenly distributed hotspots of vesicle accumulation in control neurons that are missing when hTau^0N3R^ is present (Fig. 4B). Given the noticeable lack of hot spots in the most distal pre-synaptic regions of the 6A neurons, this area was examined at 3wks (Fig. 4C–F). Levels of GFP-labelled vesicles between control and hTau^0N3R^ expressing neurons were not significantly different across pre-synaptic regions (t_(22)_ = 1.2; *P* = 0.26); but, area covered by labelled vesicles was significantly reduced (t_(21)_ = 2.5; *P* = 0.02) (Fig. 4C, D). In this region, size (t_(22)_ = 6.3; *P* < 0.0001) and number t_(22)_ = 4.2; *P* = 0.0003) of hotspots were significantly reduced when hTau^0N3R^ was present (Fig. 4E, F).

To determine timing of vesicular trafficking defects, we compared the area covered by labelled vesicles in control and hTau^0N3R^-expressing neurons for a given neuron type at 0wks using unpaired t-tests. Although 1A neurons showed an increase in area and 6B a decrease, neither were significant post correction for multiple comparisons (Fig. S8).

#### Mitochondrial trafficking

Tau over-expression causes synaptic dysfunction as a consequence of fewer functional mitochondria [24]. To see if mitochondrial trafficking defects correlated with morphological degeneration, the effects of hTau^0N3R^ on localisation of GFP-labelled mitochondria (mitoGFP) [18] was examined in the most vulnerable 6A neurons. At 3wks, there were noticeable differences in the subcellular appearance of mitochondria between control and tau expressing neurons (Fig. 4G). Mitochondria in axons are thin and tubular in control neurons and more globular when tau is present (Fig. 4G-1). Tau-expressing neurons showed greater mitochondrial accumulation in soma (Fig. 4G-2), with fewer and smaller mitochondria in dendrites (Fig. 4G-3) and distal presynaptic areas (Fig. 4G-4). Quantification of mitoGFP accumulation in the soma of 6A neurons found a significant increase in mitoGFP fluorescence in hTau^0N3R^ expressing neurons when compared to control neurons at 3wks (t_(16)_ = 2.4; *P* = 0.03)(Fig. 4H), with accumulation in soma evident as early as 0wks (t_(14)_ = 3.1; *P* = 0.008)(Fig. S9E). When total number and total volume of mitochondria across the entire neuron were quantified for hTau^0N3R^-expressing and control 6A neurons at 0 and 3wks, although trends were noted toward decrease and increase, respectively, these were not significant (Fig. S9A–D).

## Discussion

This work demonstrates the utility of a tractable in vivo model to study neuronal vulnerability in homogenous neuronal types with subcellular resolution. With this model we show that neurons of the fly CNS are differentially vulnerable to tau-mediated toxicity, thus highlighting the heterogenous nature of cellular responses to toxic proteins. The importance of innate factors in determining neuronal vulnerability to tau was recently reported in a single-cell RNA-sequencing study examining ~200 different neuron types [31]. Favouring resolution over scale, we report parallel findings highlighting importance of innate factors in influencing vulnerability, but in a small selection of homogenous neurons.

**Fig. 5:**
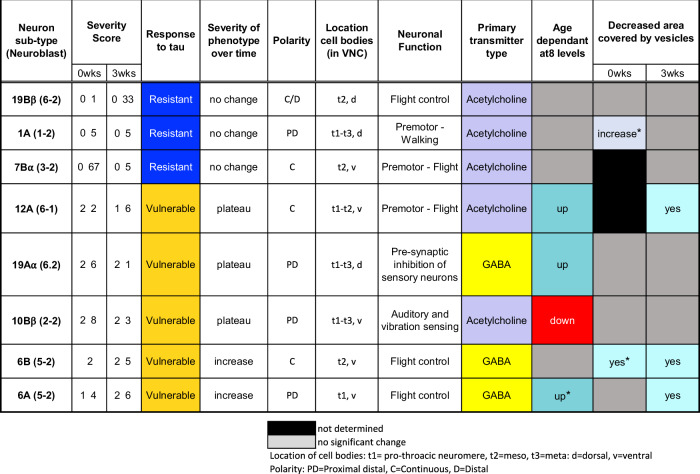
Summary of results and intrinsic characteristics of neurons examined in this study. Collated data from the literature and this study describing all eight neuron subtypes examined here. The asterisks denote observed trends not significant post correction for multiple comparisons.

Having the advantage of analysing known neurons, we sought common features of vulnerability by collating information from the literature [13, 14, 20] with noted findings and patterns between our vulnerable and resilient neurons (Fig. 5). Amongst vulnerable neuron types, there were two phases of vulnerability when determined from morphological severity scores at 0 and 3wks: during process outgrowth and elaboration of postmitotic adult neurons (during late pupariation) [32], and age-dependent (across lifespan post-eclosion). Differential effects of tau during these phases is not clear; but, variation between vulnerable neuron types suggests innate factors influence outcome. Roussarie et al. [33] identified vulnerability modules associated with AD pathology involving processes like axogenesis, microtube organisation, regulation of synaptic plasticity and neurotransmitter secretion. Thus, the first phase of vulnerability during late pupariation, may be more susceptible to disruption of these processes than the second. Why phase of vulnerability varies across neurons could depend on how quickly tau becomes toxic within a particular neuron type. Differential expression of tau toxicity modifiers across resilient and vulnerable neuron types may explain this in part [31]. Certainly, the outcome would be compounded by another factor—which tau isoform is involved. Here we expressed 0N3R tau in our neurons, which is the foetal isoform expressed during neurogenesis and the isoform that predominantly accumulates in Pick’s Disease [34]. It is possible that some property of 0N3R may have facilitated a more disruptive role during the first phase of vulnerability in some neuron types. Whether 4R tau isoforms would produce the same pattern of vulnerability remains to be seen.

Whilst all resilient types were cholinergic, vulnerable types were GABAergic or cholinergic. It is difficult to correlate transmitter type with vulnerability given our small sample size; however, Praschberger et al. noted vulnerable and resilient neurons were of mixed transmitter types [31]. Surprisingly, there also was no correlation between neuron function and vulnerability although neurons with high metabolic demands (i.e., flight associated) were expected to be more vulnerable [35].

Mechanistically, morphological vulnerability correlated well with significant age-dependent increases in pathological tau phosphorylation at Ser^202^ and Thr^205^ (AT8) sites in the same single-neuron type. Whilst our resolution is novel, age-dependant tau hyperphosphorylation is supported by the literature [36–38]. We also found phospho-tau levels vary across *Drosophila* cell types [8], and show this occurs at more spatially refined levels perhaps indicative of differential expression of tau modifying kinases or phosphatases across single-neuron types.

Vulnerability also correlated with vesicular trafficking defects which occur downstream of tau phosphorylation [23]. However, the link between p-tau levels and vesicular trafficking is not fast as 19Aα neurons showed an increase in p-tau levels over time without trafficking defects, while 6B neurons showed the opposite pattern of trafficking defects without increased p-tau. It is possible the increase in p-tau levels in 6B neurons occurred more rapidly and was missed, since 6B neurons had a high severity score and signs of vesicular trafficking defects at 0wks. Alternatively, hTau^0N3R^ could be phosphorylated at a different sites affecting microtubule stability not explored here [39, 40]. The pathway leading to degeneration in 19Aα neurons is less clear and does not seem to involve overt vesicular trafficking.

In vulnerable 6A neurons, vesicular trafficking defects at 3wks extend to presynaptic regions where hTau^0N3R^ presence results in smaller and reduced numbers of vesicular hotspots. This may be due to morphological defects or degeneration (e.g. shape and number of active zones) as the consequence of tau overexpression, but could be in part due to the effects of tau on acute vesicular localisation at presynaptic terminals [24, 41]. In the same region and timepoint, organelle trafficking issues are also observed. We noted in 6A neurons, differences in the appearance of mitochondria across subcellular compartments, and fewer mitochondria in presynaptic regions. Similar mitochondrial trafficking defects have been noted in the *Drosophila* neuromuscular junction with subsequent bioenergetic consequences for neuronal function, indicating this may represent a common mechanism of tau-mediated dysfunction [24]. Interestingly, indications of mitochondrial trafficking issues are evident in 6A neurons at 0wks, before elevated p-tau levels and significant vesicular trafficking defects. Other studies have shown tau overexpression results in mitochondrial mislocalisation to the soma by disrupting kinesin-mediated anterograde transport whilst leaving dynein-mediated retrograde relatively undisturbed, and that this can occur without affecting vesicular transport [40, 42]. This temporal glimpse into cellular events in 6A suggests mitochondrial trafficking deficits may occur earlier in disease pathogenesis before vesicular trafficking defects and overt morphological degeneration, at least in some neurons.

The appearance and mislocalisation of tau was another factor correlating with vulnerability. Tau was generally fragmented in regions correlating with morphological damage, which occurred with greater frequency in vulnerable neuron types. This supports the general belief that as tau becomes toxic, as with increased phosphorylation, it aggregates and causes degeneration. Whilst we cannot say that tau aggregates more in vulnerable neurons because we did not use aggregation-sensitive probes (e.g., Thioflavin T), the fragmentation of tau and appearance of beading on axonal processes is suggestive of tau accumulating as a precursor of aggregation. However, it should be noted that tau can cause degeneration even in the absence of filamentous aggregate formation in *Drosophila* [15, 16]; therefore, while vulnerability could be caused by an increased propensity for tau to aggregate in vulnerable neuron types, it is possible that degeneration occurs without the need for aggregation.

In vulnerable neurons, there was an age-dependent mislocalisation of tau into the finer neuronal processes of dendrites. Given that some surveyed neuron types lacked defined, purely dendritic regions suggests basic neuron anatomy (e.g. proximal-distal, continuous) may play some role in conferring vulnerability. The most affected neuron type, 6A, was polarised with large, purely dendritic regions; whereas, the most resilient,19Bβ, was mainly axo-dendritic. While not a fast rule, most vulnerable types were polarised (e.g., 6A, 19Aα, 10Bβ) and most resilient axo-dendritic (19Bβ, 7Bα). It is possible the effects of tau toxicity, such as mislocalisation of tau or organelles through microtubule dependent transport, have greater potential to damage cell types that must maintain overt cell polarity [40]. However, the relationship between anatomy and vulnerability is more complex, since neurons can be polarised and resilient like 1A. Leng et al. [43] found that morphology alone was insufficient to determine neuronal vulnerability as both excitatory large multipolar and pyramidal cells in the entorhinal complex were differentially affected in AD, rather vulnerability better correlated with the presence/absence of the gene RORB. This indicates in the very least, morphology must be coupled with a genetic propensity for vulnerability.

This latter idea is key as differential gene expression establishes neuron identity and maintains it across lifespan [44–46]. In fact, different neuron subtypes here can be differentiated by the expression of neuron subtype specific genes [14]. Thus, each neuron has a unique molecular landscape, and a cell’s capacity to cope with abnormal protein accumulation must be viewed in light of how custom contexts first determine if tau becomes dysfunctional, then, how the system deals with the ensuing stress [33, 35, 47–50]. In a simple scenario, vulnerability could arise from the presence/absence of tau modifying kinases and phosphatases within a neuron type; however, it is more likely to be the sum of many factors (e.g., levels of chaperone proteins facilitating aggregation, waste removal proteins, mechanisms for reducing oxidative stress, genes associated with neuron excitability or Ca^2+^ homeostasis, etc.) [31, 33, 47, 50].

In the context of disease, evidence from mammalian and fly RNA-sequencing studies highlight cell-type specific transcriptional changes as the consequence of normal aging and in response to AD pathogenesis and progression [44, 49, 51, 52]. These divergent and evolving landscapes make it no surprise that different neuron subtypes fall along a vulnerability spectrum, or that vulnerable neuron types show variation in the cellular events preceding neurodegeneration. There may be many routes to the same end.

Although we limited our analysis to a few tau-dependent mechanisms, our model facilitates study of a vast number of cellular processes given the plethora of available fly genetic tools. In future this model could elaborate a more holistic picture of the differential temporal and spatial events preceding neuronal degeneration in vulnerable and resilient neurons in AD and other neurodegenerative diseases.

## Supplementary information

Supplementary Figures and Tables

## Data Availability

Data is available on request.
