# Supplementary material for: Examining the vulnerability of adult neuron subtypes to tau-mediated toxicity in Drosophila
Source: Transl Psychiatry. 2025 Apr 5;15:127. doi: 10.1038/s41398-025-03342-2 (PMC11972385; doi:10.1038/s41398-025-03342-2)
Supplement: Supplementary file 1 — Supplementary Figures and Tables [file 41398_2025_3342_MOESM1_ESM.pdf]

SUPPLEMENTARY FIGURES

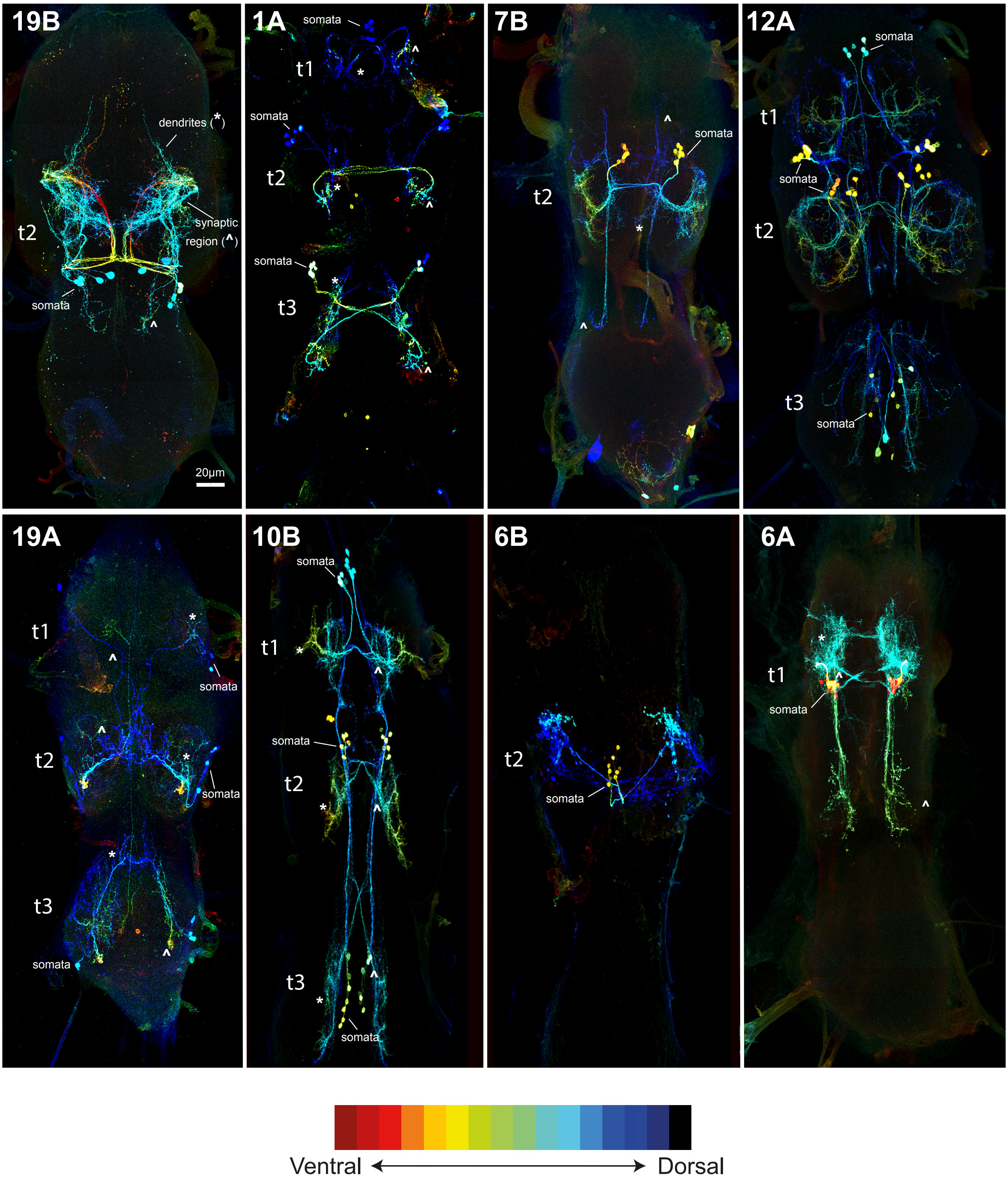

Figure S1. Characteristic cell morphology at 0wks is shown for the eight ventral nervous system (VNS) neuron hemilineages (19Bβ, 1A, 7Bα, 12A,19Aα, 10Bβ, 6B and 6A) closely examined in this study. Where some hemilineages are represented in all neuromeres, some are specific to one. Maximum intensity projections have been colour-coded to reflect pixel depth, with red presenting ventral layers (i.e., ventral anatomical structures) to blue representing more dorsal layers (i.e., dorsal anatomical structures). Structural regions with clear polarity originating off a specified somata are labelled with asterisks denoting dendritic regions (*), and carets denoting pre-synaptic regions off of axons (^).

Axodendritic arbours are unlabelled. 12A and 6B are largely axo-dendritic with little to no polarised regions.

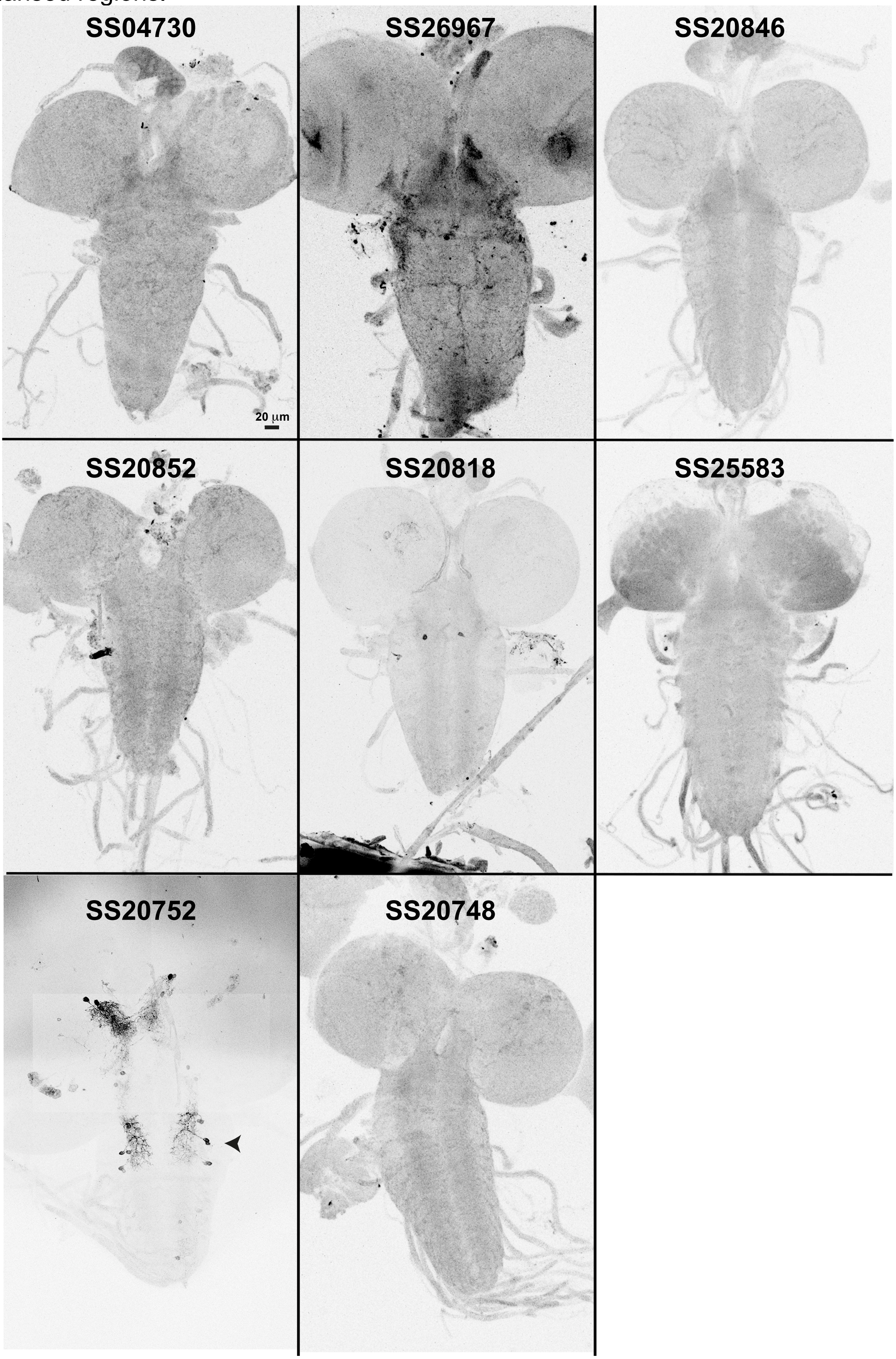

Figure S2. Patterns produced by Split-GAL4 (SS lines) driven expression of UAS-mCD8-GFP in third instar larva. Only one driver line, SS20752, shows an expression pattern in

larval brains; however, the neurons labelled at this stage in the VNC (arrowhead) are not 19A neurons.

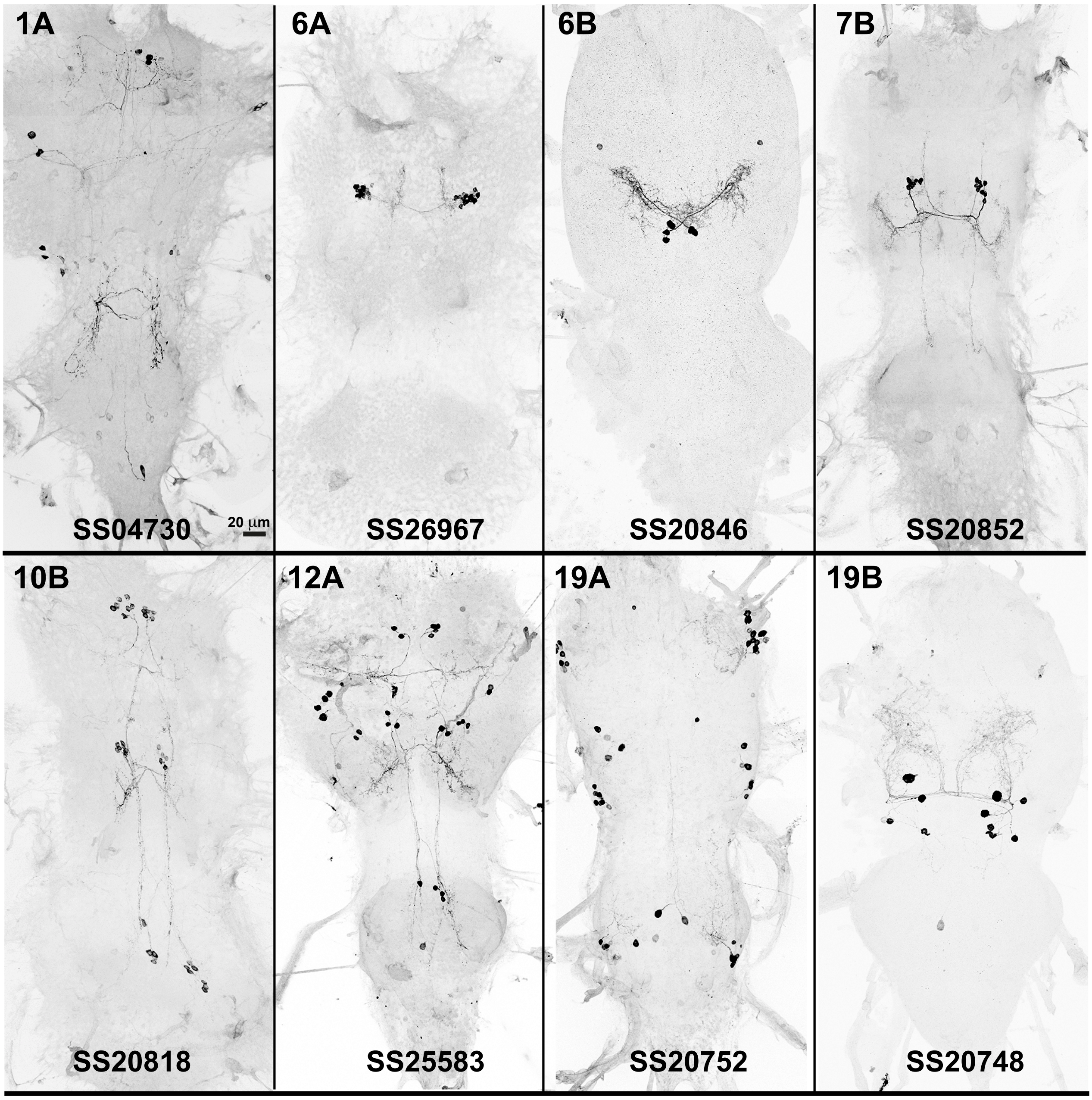

Figure S3. All drivers used in this study are on at the P8 stage of metamorphosis. The pattern produced by Split-GAL4 driven expression of UAS-mCD8-GFP in the adult fly brain is shown for each of the neuron hemilineages used in this study.

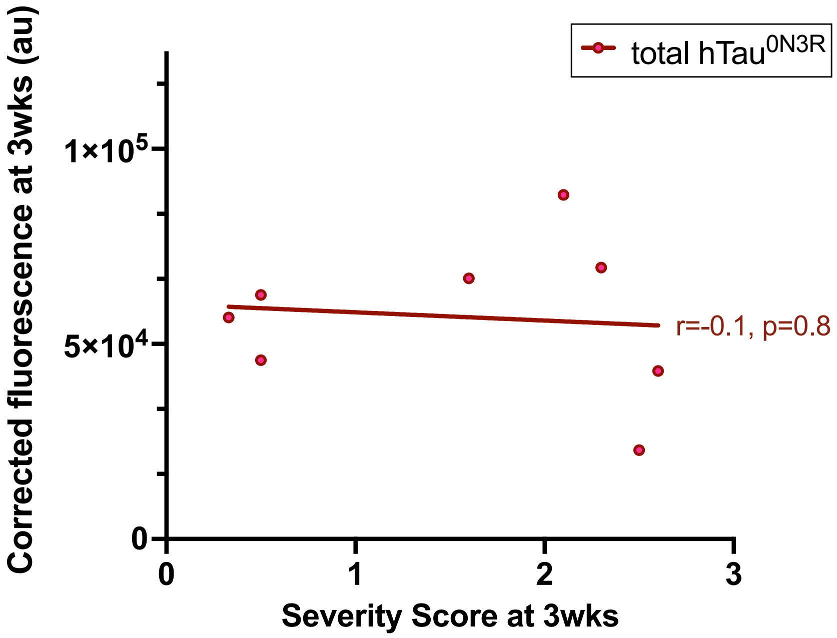

Figure S4. Morphological severity across different neuron types does not correlate with total $hTau^{0N3R}$ levels. There is no correlation between degenerative severity scores at 3wks and total tau levels at 3wks ($r=-0.10$, $p=0.8$). This indicates that morphological differences across different neuron types cannot simply be explained by differences in levels of total tau.

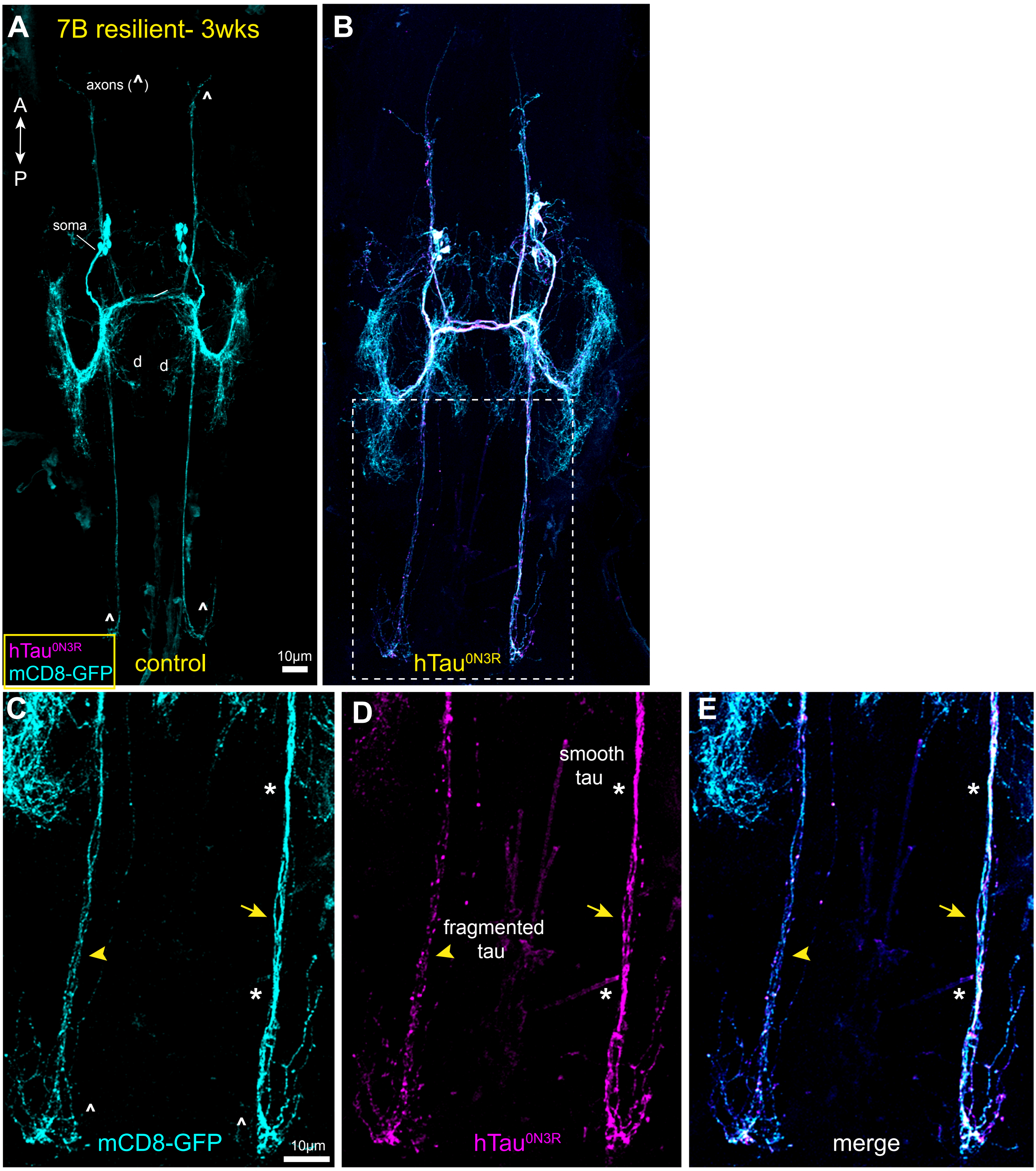

Figure S5. 7B neurons at 3wks demonstrate a relationship between tau appearance and neuron morphology. A. Control 7B neurons at 3wks appear normal. These neurons have continuous morphology and are axo-dendritic. Axons (^) are indicated, and dendrites (d) are the finer processes ipsilateral to the soma. B. Neurons expressing tau show some moderate signs of degeneration at 3wks. The area enclosed by the perforated box is enlarged in C-E. Arrowheads indicate regions containing fragmented tau colocalising to regions that show defasiculation and membrane fragmentation. Asterisks indicate regions that have continuous tau that colocalise to regions with no degeneration. Arrows point to regions showing early signs of tau fragmentation and cellular degeneration.

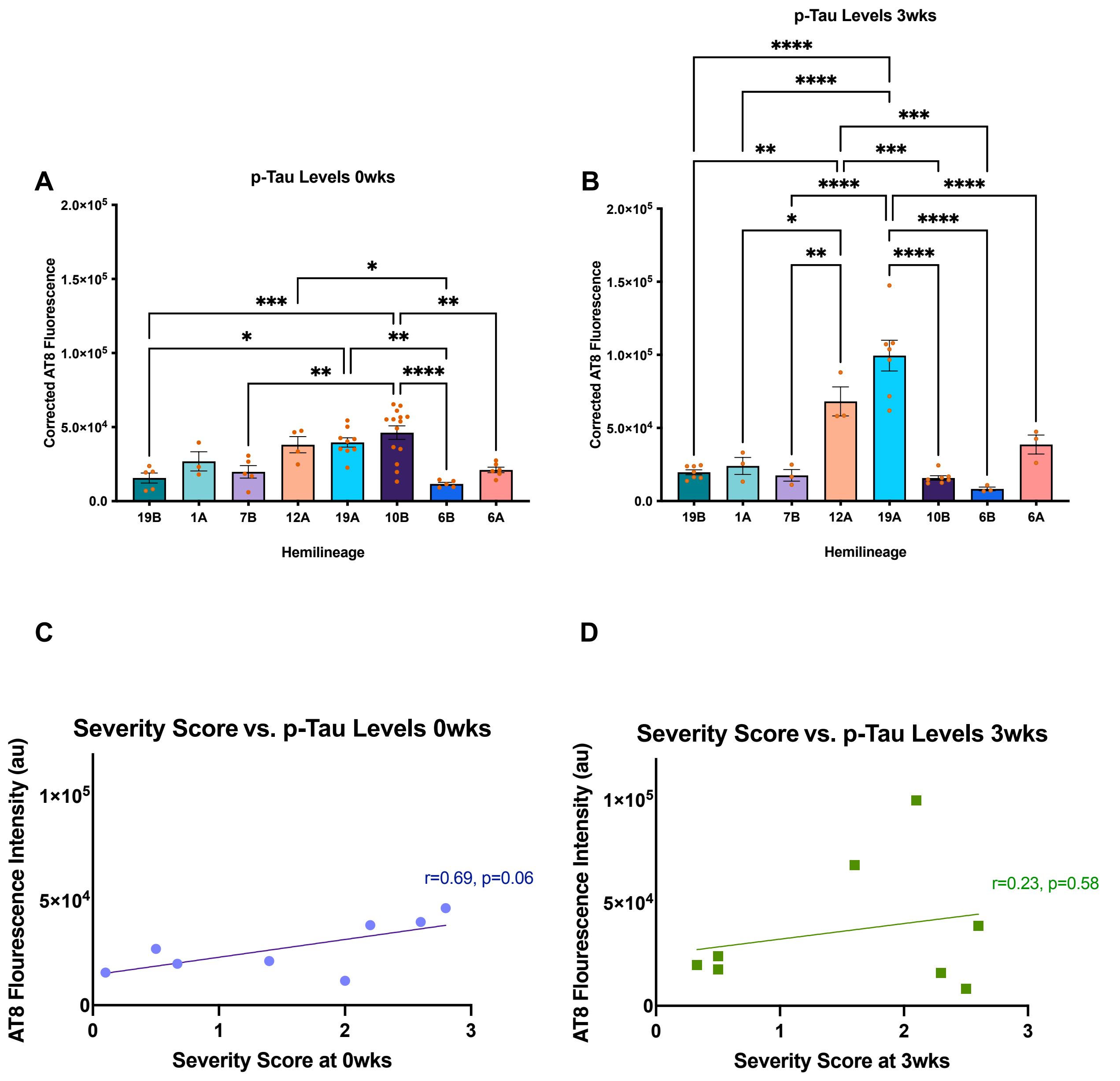

Figure S6. The role of tau phosphorylation in differential vulnerability was examined by measuring levels of tau phosphorylation at the AD-pathology associated AT8 epitope site (pS202/pT205) in hTau0N3R. Simple main effects analysis showed that time has a significant effect on p-tau levels ($P=0.0042$). Further analysis of this complex data was carried out to better understand which factors best influence vulnerability. Whilst different neuron types at a single timepoint show many significant differences in their p-tau levels (0wks: **A**, 3wks: **B**), p-tau levels do not correlate with time-matched morphological severity scores at 0wks ($r=0.69$, $p=0.06$) (**C**) or 3wks ($r=0.23$, $p=0.58$) (**D**). Thus, neuron type at a particular time point cannot provide an explanation for the observed vulnerability.

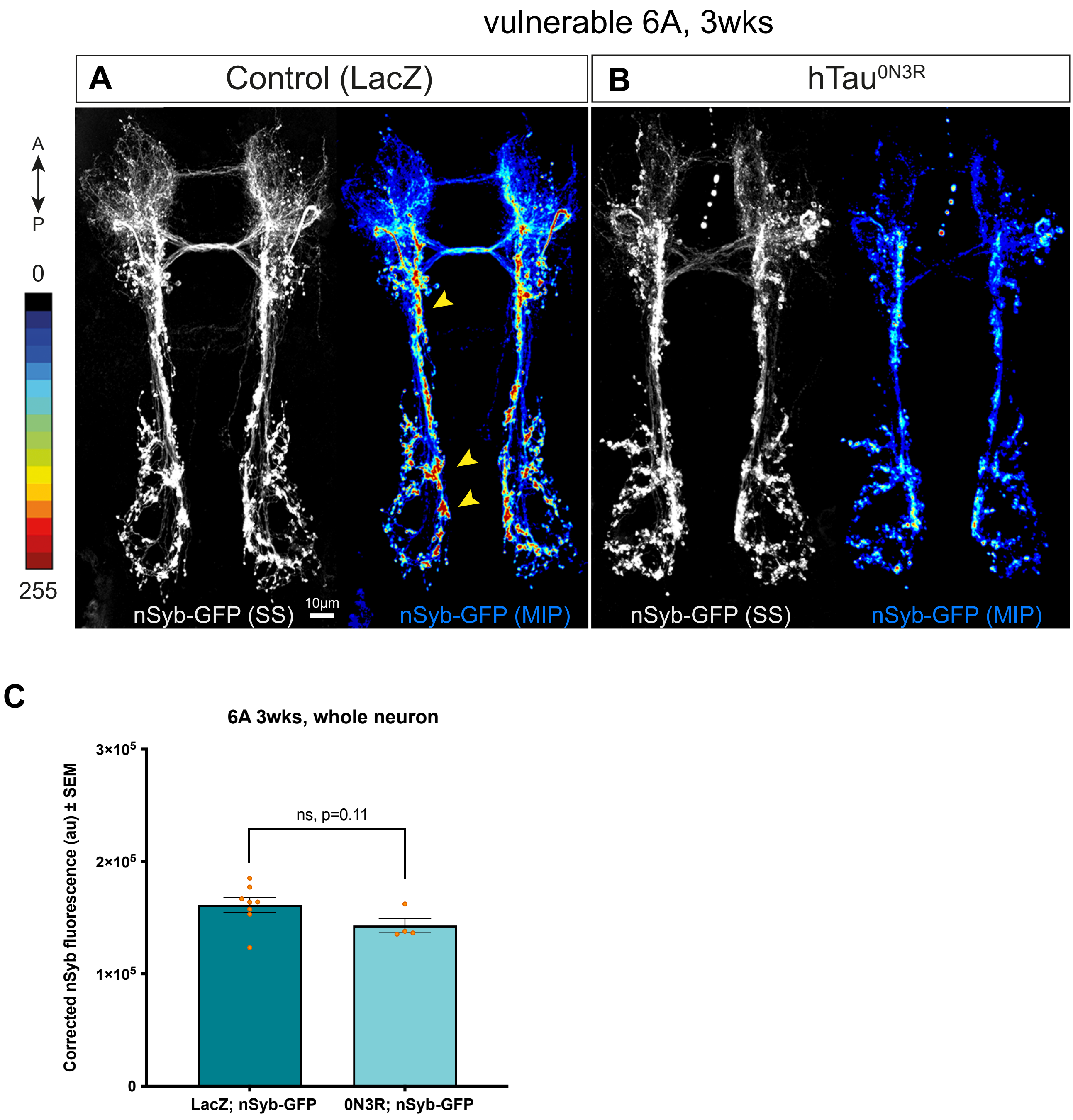

Figure S7. Despite noted trafficking issues in vulnerable 6A neurons at 3wks, total levels of GFP-labelled vesicles (nSyb-GFP) do not change between control and tau-expressing neurons. nSyb-GFP expression patterns at 3wks for control (**A**) and hTau0N3R-expressing (**B**) vulnerable 6A neurons are depicted in two ways as sum-slice (SS) showing sum of all pixel values present and thus better representing total fluorescence levels, and maximum intensity projections (MIPs) with heatmaps where pixel intensity is correlated with a colour scale. Whilst heatmaps of nSyb-GFP expression in 6A neurons reveal evenly distributed hotspots of nSyb-GFP accumulation (red) in control neurons that are lacking in hTau^{0N3R}-expressing neurons, the overall distribution of nSyb-GFP which can be viewed in the SS projections is less striking between control and tau expressing cells. The total nSyb-GFP fluorescence levels across the entire neuron are also not significantly different between control and tau-expressing neurons (**C**).

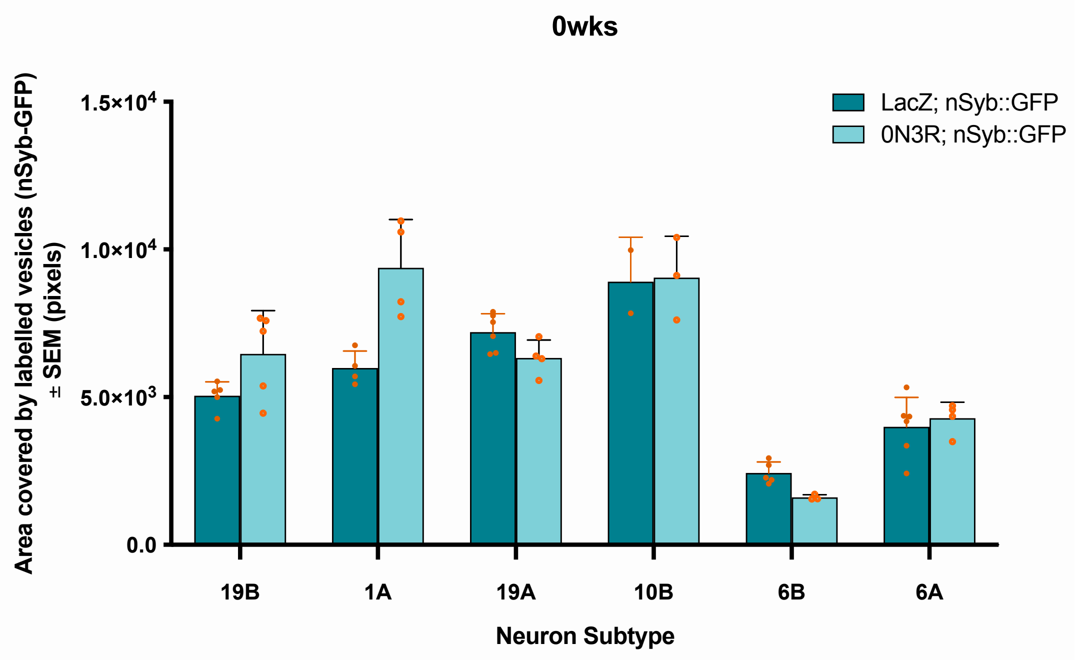

Figure S8. Vesicular trafficking at 0 weeks was examined by comparing the area covered by GFP-labelled vesicles (nSyb-GFP) between control and $hTau^{0N3R}$ expressing neurons. Whilst 1A (resilient) and 6B (vulnerable) neurons showed an increase and decrease, respectively, in the area covered by GFP labelled synaptic vesicles (nSyb-GFP), neither were significant post correction for multiple comparisons.

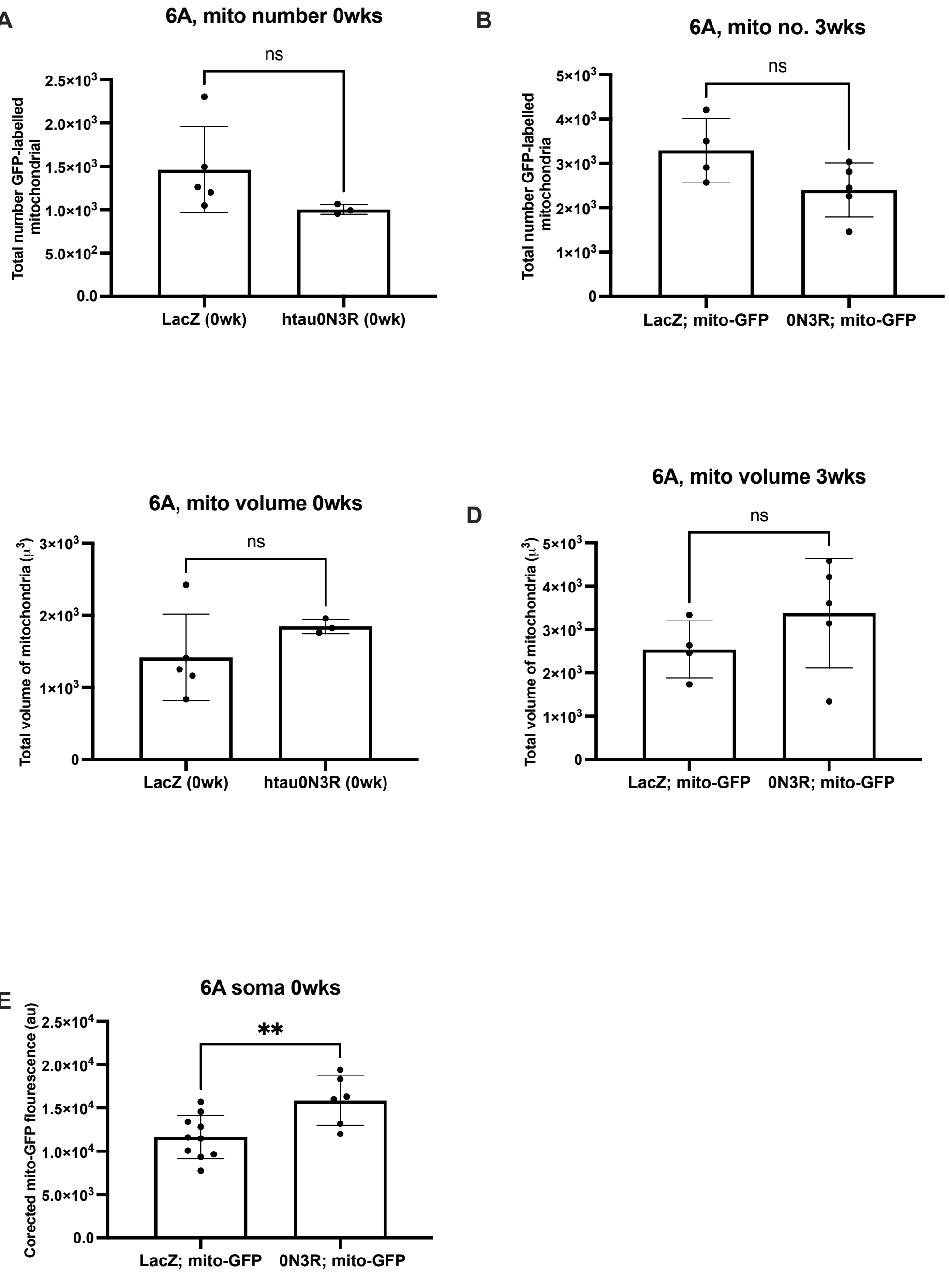

Figure S9. Mitochondrial trafficking defects were examined in the most vulnerable neuron type, 6A. **(A-D)** Between htau^{0N3R} expressing and control 6A neurons at both 0 and 3wks, although the total number and total volume of mitochondria showed trends towards decrease and increase, respectively, these are not significant. (**E**) Examination of mito-GFP levels in just the soma of 6A neurons, show a significant increase in mito-GFP fluorescence in htau^{0N3R} expressing neurons when compared to control neurons ($t_{(14)}$=3.1; P=0.008), indicating a greater accumulation of mitochondria in soma when htau^{0N3R} is present.

SUPPLEMENTARY TABLES

Table S1. Anatomy of neuron types in this study

Hemi-lineage	Driver line	Polarity & Location	Figure no. in Shepherd *et al.* (2019)*	Description of Neuron Anatomy
**1A**	SS04730	Proximal-Distal (PD)<br><br>Thoracic neuromeres t1-t3, dorsal	Figure 6, j-r	1A hemilineage neurons are local interneurons found in all three thoracic neuromeres (t1-t3). They project ipsilaterally and contralaterally in leg and wing neuropils and have clear axonal regions and fine dendritic branches. Cell bodies are located on dorsal side of the VNS.<br>Function: Coordination of leg and wing.
**6A**	SS26967	PD<br>t1, ventral	Figure 5, a-f	6A hemilineage neurons are polarized with clear dendritic and axonal regions. Cell bodies are located on the ventral surface of the VNS. Although, 6A neurons are found in t1-t3, our driver labels only t1 6A neurons. In t1 the dendrites are found ipsilateral to the cell bodies. Axons cross the midline and extend dorsally intersegmentally into the tectulum on the contralateral side, terminating in t3. These neurons are primarily associated with wing neuropil.<br>Function: Flight control
**6B**	SS20846	Continuous (C)<br>t2, ventral	Figure 5, g-k	6B neurons are largely axo-dendritic; finer processes are dendritic. Cell bodies are located on the ventral surface of the VNS. They project medially to cross the midline and arborize on either side of the midline in the tectulum. These neurons are primarily associated with wing neuropil.<br>Function: Flight control
**7Bα**	SS20782	C<br>t2, ventral	Figure 8, a-e	7B neurons are mixed axo-dendritic. Cell bodies are located on the ventral surface of the VNS, anterior of the neuromere in

				mid-ventral position. Primary neurites project dorsally and medially, crossing the midline to arborise on either side. Dendrites are finer processes ipsilateral to the cell bodies.<br>Function: Coordination of leg and wing.
**10Bβ**	SS20818	PD<br>t1-t3, ventral	Figure 3, j-n	10B are polarized intersegmental interneurons, found in all three adult thoracic neuromeres, that arborise in the medial ventral association center and intermediate neuropil. Cell bodies are located on the ventral surface of the VNS. Dendrites are confined to small dense input regions. Axonal regions are typically contralateral and in adjacent neuromeres. These neurons are primarily associated with leg neuropil.<br><br>Function: Auditory and vibration sensing. Possibly also coordinating sensory inputs between different legs.
**12A**	SS25583	C<br>t1-t2, ventral	Figure 10, a-l	12A neurons are entirely axo-dendritic. Cell bodies are located on the ventral surface of the VNS. In the prothorax, In the prothorax and metathorax, these neurons enter the neuropil and project dorsally and laterally before turning medially.<br><br>Function: Motor feedback, inhibitory back onto sensory.
**19Aα**	SS20752	PD<br>t1-t3, dorsal	Figure 12	19A neurons are polarised local interneurons with clearly defined axonal regions and fine dendritic branches. Cell bodies are located on the posterior dorsolateral side of the VNS in each segment. These neurons project arbours into the ventral leg neuropil and to a midline convergence point just below the tectulum.<br><br>Function: Sensory input from leg.

**19Bβ**	SS20748	C<br>t2, dorsal	Figure 12	19B neurons are largely axonal, with small dendritic regions. Cell bodies around found on the dorsal surface of the VNS. 19B neurons enter the neuropil and produce an arcing projection that crosses the midline and returns to the contralateral dorsal side. The primary projection produces distinct secondary projections that mostly arborise in the tectulum.<br>Function: Flight control.

*Corresponding figure detailing anatomy of a particular neuron type in Shepherd *et al.* JCM (2019)

Table S2. Criteria for Blind Scoring of morphological severity of neuron hemilineages.

Score	Criteria
0	Overall neuron morphology is normal
1	Minor: Some spottiness in dendrites and axons, some swelling of axons and major processes, lack some finer processes, spotting in finer processes
2	Moderate: Swellings in dendrites, swollen or spotty axonal endings, fragmentation, slight to some defasciculation, swollen boutons
3	Major: Loss of processes, spotty dendrites and blobby axonal endings, broken finer processes, evident axon defasciculation, axons discontinuous, loss of branches, swollen dendrites and some fragmentation, blebby axons and dendrites, loss of axonal processes

Table S3. Details of statistical and post-hoc tests used in this paper (divided by in-text and supplementary figures)

Comparison	Figure	Statistical test and Result	Post-hoc test
Tau levels between neuron types at 0wks	3A	Brown-Forsythe and Welch ANOVA test<br>F(7,13.61)=1.70, P=0.19	n/a
Tau levels between neuron types at 3wks	3B	One-way ANOVA<br>F(7,33)=11.37, P<0.0001	Tukey Test<br>1Avs6B P=0.004<br>6Avs19A P<0.001<br>6Bvs19A P<0.0001<br>6Bvs12A P=0.003<br>6Bvs19A P<0.0001<br>6Bvs19B P=0.004<br>7Bvs19A P<0.001<br>19Avs19B P=0.001
Analysis of Time (0 versus 3 wks) and neuron type on phospho-tau (AT8) levels	3C	2-way ANOVA<br><br>Interaction (Neuron-type*Time)<br>F(7,71)=16.36, P<0.0001<br><br>Neuron-type<br>F(7,71)=27.59, P<0.0001<br><br>Time<br>F(1,71)=8.74, P=0.004	Unpaired t-tests to examine phospho-tau levels over time for a particular neuron type (2C). One-way ANOVAs to examine phospho-tau levels across different neuron types at 0wks (S4A) and 3wks (S4B).
Phospho-tau (AT8) levels over time (i.e., 0 versus 3 wks data for each neuron type)	3C	Unpaired t-tests, Sidak method for multiple comparisons<br>Hemilinage P value<br>1A >0.99<br>6A 0.38<br>6B >0.99<br>7B >0.99<br>10B **<0.0001**<br>12A **0.03**<br>19A **<0.0001**<br>19B 0.99	n/a
nSyb data at 3wks<br><br>Comparison between control (beta-gal expressing) and experiment	4A	Unpaired t-tests with correction for multiple comparisons<br>Hemilinage P value<br>1A 0.76<br>6A **<0.0001**<br>6B **0.005**	n/a

($hTau^{0N3R}$ expressing) neurons at 3 wks for area covered by labelled vesicles.		7B 0.05<br>10B 0.33<br>12A **0.002**<br>19A 0.23<br>19B 0.05	
nSyb hotspots 6A synapses<br><br>Comparison between control (beta-gal expressing) and experiment ($hTau^{0N3R}$ expressing) 6A neurons at 3wks for size and number of GFP-labelled vesicles in most distal pre-synaptic regions	4C-F	Unpaired t-tests<br>6A synapses P value<br>Levels 0.26<br>Area **0.02**<br>Number **<0.0001**<br>Size **<0.001**	n/a
Corrected total mito-GFP fluorescence in 6A soma at 3wks<br><br>Comparison between control (beta-gal expressing) and experiment ($hTau^{0N3R}$ expressing)	4H	Unpaired t-tests<br>Hemilinage P value<br>6A (3wks) **0.03**	n/a
**SUPPLEMENTARY FIGURES**			
Severity score at 3wks versus total hTau levels at 3wks	S2	Correlation<br>R=-0.10, P=0.80	n/a
Phospho-tau (AT8) levels across different neuron types at 0wks	S6A	One-way ANOVA<br>F(7,43)=8.84, P<0.0001	Tukey Test<br>6Avs10B P=0.002<br>6Bvs12A P=0.03<br>6Bvs19A. P=0.002<br>6Bvs10B P<0.0001<br>7Bvs10B P=0.002<br>19Bvs19A P=0.01<br>19Bvs10B P<0.0001

Phospho-tau (AT8) levels across different neuron types at 3wks	S6B	One-way ANOVA<br>$F(7,28)=26.24$, $P<0.0001$	Tukey Test<br>1Avs12A P=0.02<br>1Avs19A P<0.0001<br>6Avs19A P<0.0001<br>6Bvs12A P=0.001<br>6Bvs19A P<0.0001<br>7Bvs12A P<0.001<br>7Bvs19A P<0.0001<br>12Avs10B P<0.001<br>19Avs10B P<0.0001<br>19Bvs12A P=0.001<br>19Bvs19A P<0.0001
Severity score at 0wks versus phospho-tau levels at 0wks	S6C	Correlation<br>R=0.69, P=0.06	n/a
Severity score at 3wks versus phospho-tau levels at 3wks	S6D	Correlation<br>R=0.23, P=0.60	n/a
Total corrected nSyb fluorescence across the entire 6A neuron between beta-gal expressing (control) and $hTau^{0N3R}$ expressing neurons at 3wks	S7C	Unpaired t-test<br>$t_{(10)}=1.8$; $P=0.11$	n/a
nSyb data at 0wks Comparison between control (beta-gal expressing) and experiment ($hTau^{0N3R}$ expressing) neurons at 0 wks for area covered by labelled vesicles.	S8	Unpaired t-tests<br><u>Hemilinage</u> <u>P value</u><br>1A 0.008*<br>6A 0.62<br>6B 0.01*<br>10B 0.92<br>19A 0.06<br>19B 0.07<br>*These comparisons do not survive correction for multiple t-tests	n/a
Comparison in 6A neurons of average total number of mitochondria, total	S9A-E	Unpaired t-tests<br>6A<br><u>Measure</u> <u>P value</u><br>Total Number:	

mitochondrial volume at 0 and 3 wks and total mitoGFP fluorescence levels in 6A soma at 0wks between Control (beta-gal expressing) and experiment (hTau^{0N3R} expressing).		0wks 0.17<br>3wks 0.08<br>Total Volume:<br>0wks 0.28<br>3wks 0.27<br>Total mitoGFP levels: 0wks **0.008**	
